# Do Altmetrics Work? Twitter and Ten Other Social Web Services

**DOI:** 10.1371/journal.pone.0064841

**Published:** 2013-05-28

**Authors:** Mike Thelwall, Stefanie Haustein, Vincent Larivière, Cassidy R. Sugimoto

**Affiliations:** 1 School of Technology, University of Wolverhampton, Wolverhampton, United Kingdom; 2 École de bibliothéconomie et des sciences de l’information, Université de Montréal, Montréal, Québec, Canada and Science-Metrix Inc., Montréal, Québec, Canada; 3 École de bibliothéconomie et des sciences de l’information, Université de Montréal, Montréal, Québec, Canada and Observatoire des sciences et des technologies, Centre interuniversitaire de recherche sur la science et la technologie, Université du Québec à Montréal, Montréal, Québec, Canada; 4 School of Information and Library Science, Indiana University Bloomington, Bloomington, Indiana, United States of America; Max Planck Society, Germany

## Abstract

Altmetric measurements derived from the social web are increasingly advocated and used as early indicators of article impact and usefulness. Nevertheless, there is a lack of systematic scientific evidence that altmetrics are valid proxies of either impact or utility although a few case studies have reported medium correlations between specific altmetrics and citation rates for individual journals or fields. To fill this gap, this study compares 11 altmetrics with Web of Science citations for 76 to 208,739 PubMed articles with at least one altmetric mention in each case and up to 1,891 journals per metric. It also introduces a simple sign test to overcome biases caused by different citation and usage windows. Statistically significant associations were found between higher metric scores and higher citations for articles with positive altmetric scores in all cases with sufficient evidence (Twitter, Facebook wall posts, research highlights, blogs, mainstream media and forums) except perhaps for Google+ posts. Evidence was insufficient for LinkedIn, Pinterest, question and answer sites, and Reddit, and no conclusions should be drawn about articles with zero altmetric scores or the strength of any correlation between altmetrics and citations. Nevertheless, comparisons between citations and metric values for articles published at different times, even within the same year, can remove or reverse this association and so publishers and scientometricians should consider the effect of time when using altmetrics to rank articles. Finally, the coverage of all the altmetrics except for Twitter seems to be low and so it is not clear if they are prevalent enough to be useful in practice.

## Introduction

Although scholars may traditionally have found relevant articles by browsing journals, attending meetings and checking correspondence with peers, in the era of digital sources they may rely upon keyword searches or online browsing instead. Whilst desktop access to many digital libraries and indexes provides potential access to numerous articles, scholars sometimes need strategies to help them to identify the most relevant articles from amongst large sets. In response, Google Scholar orders search matches in approximately decreasing order of citation, presumably with the assumption that more highly cited articles are more likely to be important or useful. Digital libraries with citation indexes often offer the same service (e.g., ACM, IEEE). In addition, digital libraries typically offer options to either sort search results by date or to confine the results to a specific year. Presumably, many scholars remain current in their fields and are therefore only interested in recent articles. However, given that citations need time to accrue, they are not the best indicator of important recent work. In response, some publishers have turned to altmetrics [1], [2], which are counts of citations or mentions in specific social web services, because they can appear more rapidly than citations. For example, it would be reasonable to expect a typical article to be most tweeted on its publication day and most blogged within a month of publication. Hence, social media mentions have become a valuable marketing tool for publishers trying to promote current high impact articles and there are also a number of altmetric tracking websites that offer free and paid services (e.g., altmetric.com, impactstory.org, and sciencecard.org).

The fact that citations take time to accumulate also has an impact on research evaluation, as a wait of a few years after publication is needed before the impact of papers can be measured (more in some disciplines). As a result, many have turned to Journal Impact Factors as a proxy for the potential citation value of articles within journals; however, due to the skewness of citation distributions [3], journal measures should not be used as article-level indicators [4]. Additionally, the relationship between citations and the Impact Factor is weakening [5]. Social media mentions, being available immediately after publication—and even before publication in the case of preprints—offer a more rapid assessment of impact. Lastly, citations only assess the impact of scholarly literature on those who cite—this neglects many other audiences of scholarly literature who may read, but do not cite (see the notion of “pure” readers [6]–[8]). In particular, the societal impact of research may not be well addressed by citations and a range of alternative methods have been developed to assess this [9]. Since the social web is widely used outside of science, it may have the potential to inform about societal impact.

The use of altmetrics in information retrieval and research evaluation begs the question: How are altmetric and citation measures related? Do social media mentions predict or correlate with subsequent citation rates for a given article? If a correlation is found, this might suggest that altmetrics and citations measure, at least to a certain extent, the same phenomenon and that altmetrics are merely early indicators of this underlying quality. The absence of such a relationship, however, would demonstrate that altmetrics probably measure something different. Given this scenario, the quality that is measured by altmetrics should be examined in order to understand the validity of using such metrics in an evaluative manner or for information retrieval.

This paper contributes to this discussion by comparing eleven different altmetric sources with citation data for 182 to 135,331 (depending on the metric) PubMed documents published between 2010 and 2012. Specifically, this study seeks to answer the following research question: To what extent do the altmetric indicators associate with citation counts?

## Background

Employing non-citation-based metrics in the evaluation of research is not novel. Previous research has looked for correlations between traditional citations and their younger counterparts: online presentations [10], online syllabi [11], Google Scholar citations [12]–[14], Google Book citations [15], and article downloads [16]–[18]. Although webometric and electronic readership studies have tried to reflect scholarly impact in a broader sense, they have often been restricted by scalability of and access to data. As altmetrics focus on social media platforms that often provide free access to usage data through Web APIs, data collection is less problematic [19].

Several sources have been proposed as alternatives for measuring the impact of scholarly publications, such as mentions and citations in blogs, Wikipedia, Twitter or Facebook or reader counts on social reference managers and bookmarking platforms [1], [19]–[22]. Evaluations of these sources have focused on single genres or sources, such as Twitter [23]–[25], blogs [26]–27, bookmarks [28], and Wikipedia [29]. Some research has focused on a variety of indicators for a single source, such as analyses of PLoS article-level metrics (ALM), which include counts of comments, ratings, social bookmarks and blog citations to articles published in the PLoS journals [4],[30]. Reader counts from social bookmarking services and social reference managers such as Mendeley, CiteULike, BibSonomy and Connotea have also been analyzed [28], [31]–[35].

A few studies have investigated altmetrics and their relationship with traditional citation indicators. Mendeley readership counts were found to correlate moderately with citations for *Nature* (r = 0.56) and *Science* (r = 0.54; [35]), *PLoS* (r = 0.5; [36]), JASIST (r = 0.46; [31],[32]), bibliometrics publications (r = 0.45; [33]) and more strongly for articles recommended on F1000 (r = 0.69; [34]). Tweets of arXiv articles (i.e., preprints of articles in mathematics, physics, astronomy, computer science, quantitative biology, quantitative finance and statistics) associate with early citation counts [25] and tweets of the *Journal of the American of Medical Internet Association* within the same year can predict future citation counts [24]. Although these results suggest that there is a positive relationship between tweets and citations, these correlation studies have mainly covered individual elite journals and those that favour internet research. The exception, for arXiv preprints also covers a somewhat special area of scholarship: articles from quantitative research areas promoted by their authors through self-archiving.

Arguments against the value of altmetrics include the ease with which they can be manipulated and their susceptibility to skew in favour of comical or sexual titles (e.g., in February 2013 the top PLoS article (from PLOS Neglected Tropical Diseases) on altmetric.com was entitled: "An In-Depth Analysis of a Piece of Shit: Distribution of Schistosoma mansoni and Hookworm Eggs in Human Stool"). In order to obtain more robust evidence, larger scale studies are needed. Moreover, the various altmetrics have different characteristics when examined diachronically. Priem, Piwowar, and Hemminger [36] examined the distribution of social media events over time for PLoS articles, noting differences in behaviour. For example, citations, page views and Wikipedia citations tended to increase over time while CiteULike, Mendeley, Delicious bookmarks, and F1000 ratings were relatively unaffected by article ages. Other metrics contained serious flaws as changes in service and limitations of data hindered analysis—this highlights concerns over the stability of some of these indicators and the use of these indicators in longitudinal studies.

It seems that altmetrics probably capture a broad, or at least a different, aspect of research visibility and impact in comparison to citation counts. For example, non-publishing so called “pure” readers are estimated to constitute one third of the scientific community [6],[7] and these may tweet or blog articles without ever citing them. Publications also influence the development of new technologies, the daily work of professionals, teaching, and also have other societal effects [37],[38], which may also be tweeted about or discussed in the social web. Kurtz and Bollen [39] classified readers of scholarly publications into four groups: researchers, practitioners, undergraduates and the interested public. Whilst all of these might use the social web, the first group is the most likely to publish scholarly papers.

Finally, the database used in this article, PubMed, indexes biomedical papers from MEDLINE as well as life science journals and online books. It is owned by the U.S. National Library of Medicine. The MEDLINE journals are selected by a technical advisory committee run by the U.S. National Institutes of Health [40].

## Methods

The goal of the research design was to devise a fair test of whether higher altmetrics values associate with higher citation counts for articles. Previous altmetric and webometric studies have tended to correlate citations with the web metric on the assumption that since citation counts are a recognised indicator of academic impact, any other measure that correlates positively with them is also likely to associate with academic impact. Correlation tests are not ideal for altmetrics, however, because many are based upon services with a rapidly increasing uptake. In consequence, newer articles can expect, on average, to receive higher altmetric scores than older articles. Since citations also take time to accrue the opposite is true for citation counts and so without adjusting for these differences a correlation test is always biased towards negative correlations. Adjusting citation and usage windows to eliminate these biases, as done with download statistics (e.g., [16],[17],[41]), is difficult as reliable usage data is only available for recent documents for which the citation window will be too small. To avoid these issues a simple sign test was devised. For this test, each article is compared only against the two articles published immediately before and after it (within the data set used and for the same journal). Thus only articles of approximately the same age, which are similarly exposed to the same citation delay and usage uptake biases, are compared to each other. Moreover any slight advantage or disadvantage of the article published after the one tested should be cancelled out by its averaging with the equivalent advantage or disadvantage of the article published before. The test gives three possible outcomes:

- *Success*: the altmetric score is *higher* than the average altmetric score of the two adjacent articles and its citation score is *higher* than the average of the two adjacent articles OR the altmetric score is *lower* than the average altmetric score of the two adjacent articles and its citation score is *lower* than the average of the two adjacent articles.
- *Failure*: the altmetric score is *higher* than the average altmetric score of the two adjacent articles and its citation score is *lower* than the average of the two adjacent articles OR the altmetric score is *lower* than the average altmetric score of the two adjacent articles and its citation score is *higher* than the average of the two adjacent articles.
- *Null*: All other cases. Note that this includes cases where all three articles are uncited, which is likely to occur when the articles are relatively new.

To illustrate the above, suppose that articles A, B, and C are ranked in publication order and attracted 2, 3, and 6 tweets respectively. Then comparing the altmetric score of B (3) with the average of the other two ((2+6)/2 = 4) results in a prediction that B will have less citations than the average of A and C. Hence if A, B, and C get 4, 6, and 12 citations respectively, then this will count as a success (as 6 is less than (4+12)/2 = 8). If they get 1, 2, and 1 citations, respectively, then this will count as a failure (as 2 is greater than (1+1)/2 = 1). If A, B and C get 1 citation each then this would count as a null result (as 1 is not greater than or less than (1+1)/2 = 1). Using the above scores, the more strongly an altmetric associates with citations, the higher the ratio of success to failure should be. Conversely, if an altmetric has no association with citations then the number of successes should not be statistically significantly different from the number of failures.

The altmetric data used originates from altmetric.com. This data was delivered on January 1, 2013 and includes altmetric scores gathered since July 2011. Although the system was undergoing development at the time and there may be periods of lost data, this should not cause false positive results due to the testing method used, as described above. The 11 metrics are the following.

- *Tweets*: Tweets from a licensed Twitter firehose are checked for citations.
- *FbWalls*: A licensed Facebook firehose is used for Wall posts to check for citations.
- *RH*: Research highlights are identified from Nature Publishing Group journals.
- *Blogs*: The blog (feed) citations are from a manually-curated list of about 2,200 science blogs, derived from the indexes at Nature.com Blogs, Research Blogging and ScienceSeeker.
- *Google*+: The Google+ Applications Programming Interface (API) is used to identify Google+ posts to check for citations.
- *MSM*: The mainstream media citation count is based on a manually curated list of about 60 newspapers and magazines using links in their science coverage.
- *Reddits*: Reddit.com posts from the Reddit API are checked for citations.
- *Forums*: Two forums are scraped for citations.
- *Q&A*: The Stack Exchange API and scraping of older Q&A using the open source version of Stack Exchange's code are used to get online questions and answers to check for citations.
- *Pinners*: Pinterest.com is scraped for citations.
- *LinkedIn*: LinkedIn.com posts from the LinkedIn API are checked for citations.

The altmetric data is not a complete list of all articles with PubMed IDs. Instead it is a list of all articles with a PubMed ID and a non-zero altmetric.com score in at least one of the altmetrics. Citations for these articles, if any, were obtained from WoS by matching the bibliographic characteristics (authors, titles, journals, and pages) of PubMed records with WoS records. First author self-citations were excluded from the results on the basis that authors would rarely hear about their work from social media. Citations and self-citations had a Spearman correlation of 0.954 for the data and so this made little difference to the results. Mentions of articles by their authors in the altmetric data were not removed because this is impractical (e.g., due to Twitter usernames not conforming to guidelines); it seems that no previous study and no altmetric web site has attempted to remove self-citations. There were 3,676,242 citations altogether to the articles in the data set, excluding self-citations. Although the citation scores for the articles are not reliable due to the short citation windows, this should not cause systematic biases in the results because publication time is taken into account in the method used to compare citations with altmetric scores.

For each journal and each altmetric, a list was created of all articles with a score of at least 1 on the altmetric, discarding articles with a zero score. The reason for the discarding policy was that the data set did not include a complete list of articles in each journal and it was impractical to obtain such a list. Moreover, since the authors did not have control over the data collection process, altmetric data for articles may be missing due to problems in the data collection process (e.g., due to the matching processes used). As a consequence of this, it is not possible to be sure that articles with zero values for an altmetric should not have positive scores (unlike [42] for example). It is more certain that articles with a positive score on an altmetric had their data effectively collected with that altmetric and so data for articles with non-zero altmetric scores is the most reliable and is the only data used in this article. Since the data collection process varies between altmetrics, it is not possible to assume that a positive score for an article on one altmetric implies that it will also have been effectively monitored for all the other altmetrics. Preliminary testing showed that this was not the case (resulting in a preliminary analysis of the data with additional implied zeros for articles with a non-zero score on one altmetric but a positive score at least one other altmetric being rejected as unreliable and not reported here). The discarding policy allowed each list to be complete in the sense of including all articles with an altmetric score >1. The results, therefore, only relate to articles attracting a positive altmetric score.

To obtain the chronological order needed for the sign test, for each journal and altmetric, the document lists were ordered by PubMed ID. Although imperfect, this was the most reliable general source of chronological information available. DOIs sometimes contain chronological information, such as a year, but even when a year is present it can refer to the submission year, acceptance year or publication year. Although the publication year and issue number are included in the bibliographic metadata, they are not detailed enough and in many cases do not reflect the actual date of online availability. In contrast, the PubMed ID is more fine-grained and universal. It seems likely to be reasonably chronologically consistent for each individual journal, if not between journals. As a validity check for this, PubMed IDs were correlated with citation scores, providing a value of −0.611. Cross-checking DOI-extracted years with PubMed IDs also confirmed that the use of PubMed IDs to represent time was reasonable. PubMed supplies a range of dates for articles, including Create Date, Date Completed, Date Created, Date Last Revised, Date of Electronic Publication, Date of Publication, and date added to PubMed and, of these, date of electronic publication would also be a logical choice for date ordering. Conducting the main analysis for journals separately ensures that predominantly articles from the same subject area are compared, except in the case of multidisciplinary journals. For journals with few articles in the data set any comparisons between altmetrics and citations are likely to be not statistically significant but it is still possible to test for statistical significance on the number of *journals* for which citations for individual articles associate positively with altmetrics more often than negatively.

A simple proportion test was used for each altmetric to see whether the proportion of successes was significantly different from the default of 0.5. Null results (i.e., neither success nor failure) were ignored because these do not represent the presence of absence of an association. The proportion of null results is irrelevant because this depends to a great extent on the time since the data was collected. For instance, almost all recent data would have zero citations recorded and would hence give a null result. The number of null results therefore reveals nothing about the long term underlying relationship between an altmetric and citations. The test can occur only for journals with at least three articles in the data set and the number of tests is 2 less than the number of articles in the journal. This accounts for the differences between the number of articles and the number of tests in Table 1. The number of journals differs between tables 1 and 2 because table 1 only includes journals with at least one non-null test. A Bonferroni correction for multiple tests was used to hold constant the probability of incorrectly rejecting the null hypothesis. For the p = 0.05 level, this reduces the p value to 0.0046 and for the p = 0.01 level, this reduces the p value to 0.0009.

**Table 1:** The number of successes and failures for comparisons of citations and metric scores for articles with non-zero metric scores.

Metric	Successes	Failures	Z	Null	Total tests	Journals	Articles
Tweets**	24315 (57%)	18576 (43%)	27.7	159242	202133	3303	208739
FbWalls**	3229 (58%)	2383 (42%)	11.3	32037	37649	1850	41349
RH**	3852 (56%)	3046 (44%)	9.7	57857	64755	1004	66763
Blogs**	1934 (60%)	1266 (40%)	11.8	20383	23583	992	25567
Google+	426 (53%)	378 (47%)	1.7	2399	3203	332	3867
MSM**	338 (59%)	232 (41%)	4.4	1651	2221	196	2613
Reddits	103 (56%)	81 (44%)	1.6	1799	1983	178	2339
Forums**	19 (86%)	3 (14%)	3.4	43	65	8	81
Q&A	12 (67%)	6 (33%)	1.4	266	284	51	386
Pinners	4 (80%)	1 (20%)	1.3	264	269	50	369
LinkedIn	0 (-)	0 (-)	-	42	42	17	76

Articles are only compared against other articles from the same journal.

* Ratio significantly different from 0.5 at p = 0.05, **Significant at p = 0.01; Bonferroni corrected for n = 11.

**Table 2:** Successes and failures for articles with non-zero metric scores, aggregated by journal, and only including journals for which there it is at least one success or failure.

Metric+	Mostly success	Mostly failure	Z	Equal	Journals
Tweets**	1097 (58%)	646 (34%)	10.8	148 (8%)	1891
**	1032 (59%)	586 (33%)	11.1	139 (8%)	1757
FbWalls**	414 (53%)	282 (36%)	5.0	86 (11%)	782
**	308 (55%)	188 (34%)	5.4	62 (11%)	558
RH	276 (51%)	221 (41%)	2.5	47 (9%)	544
	193 (51%)	157 (41%)	1.9	30 (8%)	380
Blogs**	190 (58%)	104 (32%)	5.0	32 (10%)	326
**	129 (57%)	70 (31%)	4.2	26 (12%)	225
Google+	61 (50%)	53 (44%)	0.7	7 (6%)	121
	25 (48%)	24 (46%)	0.1	3 (6%)	52
MSM	29 (56%)	17 (33%)	1.8	6 (12%)	52
	13 (52%)	9 (36%)	0.9	3 (12%)	25
Reddits	22 (51%)	17 (40%)	0.8	4 (9%)	43
	9 (47%)	7 (37%)	0.5	3 (16%)	19
Forums	5 (83%)	1 (17%)	1.6	0 (0%)	6
	3 (100%)	0 (0%)	1.7	0 (0%)	3
Q&A	4 (67%)	1 (17%)	1.3	1 (17%)	6
	2 (67%)	0 (0%)	1.4	1 (33%)	3
Pinners	2 (67%)	1 (33%)	0.6	0 (0%)	3
	0 (−%)	0 (−%)	-	0 (−%)	0
LinkedIn	0 (−%)	0 (−%)	-	0 (−%)	0
	0 (−%)	0 (−%)	-	0 (−%)	0

+ In each cell the upper figure is for all journals and the lower figure is for journals with at least 10 articles tested. * Ratio of successes to failures significantly different from 0.5 at p = 0.05, ** Significant at p = 0.01; both Bonferroni corrected for n = 11.

## Results and Discussion

In all cases except Google+ and Reddit and those for which under 20 articles were available to be tested (Q&A, Pinners, LinkedIn), the success rate of the altmetrics at associating with higher citation significantly exceeded the failure rate at the individual article level (Table 1). The null column of the table includes many cases of new articles with only one altmetric and no citations and therefore is potentially misleading because the articles may receive citations later and so the altmetric scores for the same articles could then become successes or failures. Overall, there are no cases where the number of failures is lower than the number of successes and so this suggests that, given sufficient data, all the altmetrics would also show a significantly higher success than failure rate. The case that runs most counter to the hypothesis that altmetrics associate with citations is Google+, which launched on June 28, 2011 and has non-significant results despite a large number of tagged articles. This may be a statistical anomaly since the ratio of successes to failures is only slightly above 50% for the metrics with significant scores (except for forums).

The number of *journals* for which the success rate of articles exceeds the failure rate (although not necessarily with a significant difference within a journal) is a majority in all cases for which there is sufficient data (Table 2) and the difference is significant for three cases. This result stays the same if the data is restricted to journals with at least 10 tested articles. In summary, there is clear evidence that three altmetrics (tweets, FbWalls, blogs) tend to associate with citations at the level of individual journals. Although for almost all metrics there are some journals for which the sign test produces more failures than successes, these tend to happen for journals with few articles tested and hence the majority failure could be a statistical artefact (i.e., due to normal random variations in the data). For instance, the 25 journals with the most tweeted articles all give more successes than failures. For tweets, the journal with the most articles and more failures than successes is the 26^th^, *Proceedings of the Royal Society B* (biological sciences), with 117 prediction successes and 118 failures. This difference of 1 is easily accounted for by normal random factors in the data. In contrast, the most tweeted journal, Proceedings of the National Academy of Sciences had 1069 successes and 818 failures (57% and 43%, respectively, of articles that were either success or failures), a small but significant difference. Note that the magnitude of the difference between success and failure in Table 2 is not helpful to interpret because this is primarily dependent upon the proportion of journals with few articles represented for which the chance of success or failure is nearly 50%. Similarly, the magnitude of the differences between the success and fail rates in both tables 1 and 2 are not significant due to the simple tests used, and the magnitude of the correlation in Table 3 is misleading due to the conflicting (assumed) citation association and negative time association and so the results do not shed any light on the magnitude of the association between citations and altmetric scores in the cases where an association is proven.

**Table 3:** Correlations between metric values and citations (excluding self-citations) for all articles with non-zero scores on each altmetric.

Metric	Spearman	Articles (>0)	Metric total
Tweets	−0.190**	135,331	359,176
FbWalls	0.050**	24,822	35,317
RH	0.373**	23,980	35,365
Blogs	0.201**	13,325	17,699
Google+	0.034**	3,440	5,531
MSM	0.088**	2,402	3,209
Reddits	0.062**	1,516	1,766
Forums	0.033**	82	121
Q&A	0.048**	335	372
Pinners	0.005**	301	324
LinkedIn	0.009**	171	174

* Significant at p = 0.05, ** Significant at p = 0.01; both Bonferroni corrected for n = 11.

The problem of non-significant differences between success rates and failure rates for individual journals could be avoided in Table 2, in theory, by replacing the figures in the second and third columns with the number of journals for which the difference between the number of successes and failures is statistically significant. This is not possible, however, because too few journals have enough articles tested to give a reasonable chance of a statistically significant result. Nevertheless, the results are consistent with but do not prove the hypothesis that all the altmetrics tested associate with higher citations.

Although the results are clear for most metrics, they only cover articles with a non-zero altmetric score. It is theoretically possible, but does not seem probable, that the same is not true for all articles. For the omission of articles with zero altmetric scores to bias the results towards sign test failures, articles with zero altmetric scores would need to be more cited than average for articles published at the same time that had a positive altmetric score. This seems unlikely since the results here show that increased altmetric scores tend to associate with increased citations. Another limitation is that the results are only for PubMed articles and so it is not clear whether they would also apply outside the biomedical and life sciences. The differing sample sizes for the altmetrics is also important because altmetric-citations associations may well be significant for most of the altmetric but hidden by insufficient data. Finally, unlike one previous study [24], no predictive power can be claimed from the results. Although it seems likely that most altmetric values precede citations - for example, tweets seem to appear shortly after an article has been posted online [25] - this has not been tested here because the data does not include origin dates for the scores. In other words, we did not directly test that high altmetric scores today make high citations tomorrow more likely.

Related to the issue of predictive power, it is clear from Table 1 that, other than tweets, the other metrics had a high proportion of zero scores. For instance there were only 20% as many Research Highlights articles as tweeted articles and only 0.04% as many articles in LinkedIn as tweeted articles. These figures are only estimates because there may be missing data and other data collection methods may have been able to identify more matches in all cases (including for Tweets). Nevertheless, the disparities in numbers of articles in Table 1 highlight that the coverage of the altmetrics, and particularly those other than Twitter, may be low. A low coverage in combination with statistically significant results for an altmetric suggests that it is not useful to differentiate between average articles but may only be useful for identifying either exception articles or a sample of above average articles.

Correlation tests were run on the data to test the importance of time for identifying significant associations between altmetrics and citations. Whilst four of the altmetrics significantly and positively correlate with citations (with a medium correlation effect size for RH, small for blogs, smaller for MSM and FBWalls [43]), the correlation for Twitter is significant and negative (with a small effect size [43], Table 3). The reason seems to be that Twitter use is increasing much faster than the others, so that more recent articles are more tweeted but are typically uncited. In other words, this reflects the two biases of correlation coefficients, described above, that are caused by the level of social media uptake on the one hand and that of citation delay on the other. To test this we ran another correlation test for Twitter based on articles from 2010 based upon their DOI (i.e., a very approximate heuristic since this could be the submission date, the acceptance date, the online first date or the final publication date), finding a small significantly negative correlation of -0.236. A partial correlation to remove the influence of time through PubMed IDs (again a heuristic, especially because of it being used across multiple journals that may have different PubMed submission strategies) improved this to an almost zero correlation of 0.009, tending to confirm the importance of time. An implication of these results for publishers and digital library users, is that time from publication should be considered in addition to altmetric scores when using altmetrics to rank search results.

The negative correlation from Tweets in Table 3 should not be interpreted as evidence that high tweet counts do not associate with high *quality* articles. On the contrary, the evidence from tables 1 and 2 is that tweets *are* useful at indicating more highly cited articles; the negative correlation in Table 3 is due to tweets for uncited articles that, if the trend continues, will tend to become more highly cited over time. Note that these correlations are not reliable because they include articles from multiple journals with different citation rates, with different PubMed submission times and strategies, and that are associated with fields that presumably have different cultures of Twitter use.

The correlations in Table 3 also confirm that the magnitude of the significant results in Tables 1 and 2 do not give evidence of the likely size of the underlying correlation between the altmetrics and citations. For example, there are positive associations for Twitter in Tables 1 and 2 and a negative correlation in Table 3. Hence it is not possible to speculate about the degree of accuracy for citation estimates made with altmetrics from the data set used here.

## Conclusions

The results provide strong evidence that six of the eleven altmetrics (tweets, Facebook wall posts, research highlights, blog mentions, mainstream media mentions and forum posts) associate with citation counts, at least in medical and biological sciences and for articles with at least one altmetric mention, but the methods used do not shed light on the magnitude of any correlation between the altmetrics and citations (i.e., the correlation effect size is unknown). Nevertheless, the coverage of all of the altmetrics, except possibly Twitter, is low (below 20% in all cases and possibly substantially below 20%) and so these altmetrics may only be useful to identify the occasional exceptional or above average article rather than as universal sources of evidence. The evidence also suggests that Google+ posts might possibly have little or no association with citations, and too little data was available to be confident about whether four of the metrics (LinkedIn, pinners, questions, and reddits) associate with citation counts. Nevertheless, given the positive results for the majority of metrics it would be reasonable to suppose that all may associate with citations and that if more data could be collected then this would be evident. In this case, a social web service would still need to be sufficiently used for citations to give enough data to be worth reporting or analysing (e.g., possibly not for LinkedIn, Pinners and Q&A). These results extend the previously published evidence of a relationship between altmetrics and citations for arXiv preprints and a few individual journals and two social web altmetrics (Mendeley and Twitter) to tests of up to 1,891 biomedical and life sciences journals and 11 altmetrics (6 with positive results). This study also introduced a simple method, the sign test, to eliminate biases caused by citation delays and the increasing uptake of social media platforms.

Another important finding is that because of the increasing use of the social web, and Twitter in particular, publishers should consider ranking or displaying results in such a way that older articles are compensated for lower altmetric scores due to the lower social web use when they were published. Without this, more recent articles with the same eventual impact as older articles will tend to have much higher altmetric scores. In practice, this may not be a significant worry, however, because those searching the academic literature may prefer to find more recent articles.

Although the results above suggest that altmetrics are related to citation counts, they might be able to capture the influence of scholarly publications on a wider and different section of their readership than citation counts, which reflect only the behaviour of publishing authors. However, more research – quantitative and qualitative – is needed to identify who publishes citations to academic articles in social web sites used to generate altmetrics (e.g., students, researchers, the general public), and why they publish them. Results in terms of user groups, users' motives and level of effort are likely to vary between social media platforms, which must be taken into consideration when applying different altmetrics in research evaluation and information retrieval.
